# The Skin Microbiome, Microbial Metabolites and the Epidermal Response to Ultraviolet Radiation—Towards Next Generation Suncare

**DOI:** 10.1111/exd.70142

**Published:** 2025-07-24

**Authors:** Steven D. Mercer, Andrew J. McBain, Catherine O'Neill

**Affiliations:** ^1^ Division of Musculoskeletal and Dermatological Sciences, Faculty of Biology, Medicine and Health, School of Health Sciences The University of Manchester Manchester UK; ^2^ Division of Pharmacy and Optometry, Faculty of Biology, Medicine and Health, School of Health Sciences The University of Manchester Manchester UK

**Keywords:** microbial metabolites, microbiome, skin health, tryptophan, ultraviolet radiation

## Abstract

Ultraviolet radiation (UVR) presents one of the greatest challenges to human skin, with numerous studies documenting its effects on skin physiology. Recently, growing recognition of the microbiome's crucial role in skin health has led to investigations on how UVR influences skin‐microbiome interactions. Research in mice suggests that the microbiome plays a key role in regulating the skin's response to UVR, impacting inflammation, immune function, and keratinocyte differentiation. These effects may be mediated by microbial metabolites (MM), yet the impact of UVR on microbial metabolism and its subsequent effects on skin health remains poorly understood. Some studies suggest that UVR exposure may modify the composition of the microbiome, which could, in turn, alter the microbial metabolome. This viewpoint reviews the current literature regarding the interplay between the skin, its microbiome, and UVR, and speculates on how UVR‐induced changes to microbial composition and metabolism might affect skin health. Furthermore, future areas of research that should be considered and the potential of MM in next generation suncare, cosmetics and therapeutics will be highlighted.

## 1 Introduction

As arguably the greatest challenge to the human skin, decades of investigation have described the effects of ultraviolet radiation (UVR) on skin physiology. More recently, with a growing recognition of the crucial role of the microbiome in skin health, studies have begun to assess the impact of UVR on skin‐microbiome interactions [1, 2, 3]. Current studies have shown that the microbiome is essential for regulating the cutaneous response to UVR exposure by moderating inflammation, immune response, and keratinocyte differentiation [2, 3, 4]. Some of these effects may be mediated by the production of microbial metabolites (MMs).

The impact of UVR on the microbiome itself remains poorly understood, with limited studies suggesting UVR exposure can alter microbiome composition and abundance [1, 5]. In this article, we argue that changes to microbial composition in response to UVR may have ‘knock‐on’ effects for the skin, through loss of important bacteria or changes to bacterial metabolism, altering production of MMs important for skin health. In addition, this article will review the current literature on the skin, its microbiome, and the influence of UVR on their interactions. Based on this evidence, we will speculate on the potential effects of UVR on the skin microbiome and its implications for skin health. Finally, we will hypothesise as to how an increased understanding of the skin‐UVR‐microbiome axis may potentially lead to development not only of new ‘suncare’ products but also new therapeutics for the photodermatoses.

## 2 The Skin Microbiome Is Essential for Epidermal Health

The human skin is inhabited by a rich microbiome, comprised of a range of bacteria, viruses, fungi and mini eukaryotes [6]. A typical human houses roughly 1000 different species on their skin [7] with each microenvironment being colonised by different bacterial genera [8]. Multiple factors can affect the composition of the microbiome, including the host, disease, and environmental factors such as cosmetics and UVR [8]. The microbiome is now known to contribute to skin health in numerous ways. For example, resident microbes can prevent the colonisation of pathogenic species by competing for space and nutrients [8] or by exhibiting direct antimicrobial properties, such as the competition between *Staphylococcal* species. Antimicrobial peptides (AMPs) released by coagulase‐negative *Staphylococci* (CoNS) can selectively kill *S. aureus* , with atopic dermatitis (AD) patients having significantly reduced colonisation of CoNS and higher *S. aureus* bioburden [9]. *S. epidermidis* can also reduce *S. aureus* colonisation in newborn infants [10]. Skin commensal bacteria are also known to influence immune cell development, with roles in educating T‐regulatory cells and developing CD169^+^ macrophages [11, 12].

Skin bacteria are also known to be critical to epidermal barrier function. Uberoi et al. [13] demonstrated that skin from germ‐free (GF—having no microbiome) mice had reduced epidermal thickness, keratin‐10 expression, and increased trans‐epithelial water loss (TEWL) when compared to pathogen‐free (PF) mice. The barrier defects were negated by the introduction of a consortium of bacteria from the human skin microbiome. These effects were mediated via the aryl hydrocarbon receptor (AHR) which is well recognised for its role in epidermal differentiation as well as other functions [14].

Lai et al. [15] reported that a molecule released by *S. epidermidis* increased the expression of human β‐defensins (HβDs: a type of AMP) in keratinocytes by interacting with toll‐like receptor (TLR) 2. Work by the same group found that lipoteichoic acid secreted by *S. epidermidis* interacts with TLR3 on keratinocytes and mediates pro‐inflammatory cytokine expression after compromise of the epithelial barrier [16]. *S. epidermidis* is also known to stimulate the production of other AMPs, including RNase7 [17] and increase trans‐epithelial electrical resistance (TEER) of keratinocytes [18]. These and many other examples point to a critical role for the microbiome in skin health [2, 3, 4, 7, 8, 19, 20, 21, 22].

## 3 The Microbiome Influences Skin Health via the Production of Metabolites

Current work suggests that the influence of the microbiome on skin physiology is due, at least in part, to the production of MMs. There are two types of MM: primary (PM) and secondary (SM). PMs are required for microbial growth and therefore have limited diversity [23]. SMs are extremely diverse and are often characteristic of certain strains or species, or the available nutrients [24, 25]. As they are not required for growth, SM production is associated with increased fitness, often acting as antifungals or antimicrobials [23, 26]. SMs encompass a wide variety of chemical classes, including non‐ribosomal peptides, polyketides, terpenoids, phenazines, alkaloids and short‐chain fatty acids (SCFA). These compounds vary significantly in size and complexity, ranging from small volatile molecules of 100–200 Da to large multifunctional structures such as cyclic peptides and polyketides over 1000 Da [24, 27]. Some SMs, such as ectoine, reuterin, lactic acid, and select bacteriocins (antimicrobial metabolites), are already used in skincare products for their antimicrobial, microbiome‐modulating, anti‐inflammatory, and barrier protective properties [28, 29, 30]. Although there is limited understanding of how MMs affect the skin, there are now several studies pointing to their importance to skin health (summarised in Table 1).

**TABLE 1:** Microbial metabolites associated with skin health.

Metabolite	Class of metabolite	Molecular weight (Da)	Metabolised from	Role in skin health	References
Lactic acid	Organic acid, PM	90.08	Fermentation of sugars by various bacteria, particularly *Lactobacillus*	Topical application and keratinocyte culture with lactic acid producing species helps maintain skin pH, promotes hydration, supports barrier function and mediates inflammation. Cosmetic formulations can also increase skin smoothness and firmness	[29, 30, 31]
Acetic acid	Organic acid, PM	60.05	Fermentation of sugars by various bacteria, particularly *Acetobacter*	Oral intake of *Acetobacter* increased the stratum corneum hydration Topical application of acetic acid inhibits pathogen growth and improves wound healing	[32, 33]
Butyric acid	SCFA (FFA), SM	88.11	Fermentation of dietary fibres by gut bacteria (and some skin bacteria like *S. epidermidis* )	Gut derived butyric acid in mice exhibits anti‐inflammatory properties and enhances skin barrier integrity Skin derived butyric acid reduces IL‐6 expression after UVR exposure	[21, 34]
Propionic acid/propionate	SCFA (FFA), SM	74.08	Fermentation of amino acids or sugars by *Cutibacterium*	Maintains skin pH and inhibits bacterial growth, contributes to the pathogenesis of *C. acnes*	[35]
Urocanic acid	Amino acid derivative/organic acid, SM	138.14	Breakdown of histidine by environmental bacteria and *S. aureus*	Absorbs UVR and supports skin barrier	[36, 37]
Hydrogen peroxide	Reactive oxygen species, SM	34.01	Oxygen reduction in bacterial metabolism. Produced by *Staphylococcus* and *Lactobacilli*	Involved in pathogen inhibition	[38, 39, 40]
Phenylacetic acid	Aromatic acid, SM	136.15	Breakdown of phenylalanine by bacteria such a *Pseudomonas*	Antimicrobial activity	[41]
Ceramides	Lipid derivative, SM	> 582	Sphingolipid and phospholipid breakdown by bacteria such as *S. aureus*	Already part of host‐derived skin composition, ceramides help maintain the skin barrier, retain moisture and restore compromised barrier	[42, 43, 44]
Nicotinamide (vitamin B3)	Vitamin/amino acid metabolite, SM	122.12	Nicotinic acid metabolism by bacteria in the gut	Topical application is involved in energy metabolism, promotes DNA repair, barrier maintenance and hydration Gut derived prevents and improves response of treatment to skin cancer	[45, 46]
Oleic acid	Fatty acid (FFA), SM	282.46	Hydrolysis of triglycerides by skin bacteria	Inhibits adhesion and biofilm formation of *S. aureus*	[47, 48]
Azelaic acid	Fatty acid (FFA), SM	188.22	Oleic acid or nonanoic acid metabolism by *Malassezia furfur*	Anti‐inflammatory and antimicrobial against numerous strains	[49, 50]
Porphyrins	Amino acid metabolite, SM	> 616.7	Breakdown of amino acid δ‐aminolevulinic acid by *C. acnes*	Absorb UVR and protect bacterial cells and surrounding tissue from UVR	[4, 51, 52]
Valerate	SCFA (FFA), SM	102.13	Breakdown of glycerol or lipids by *C. acnes*	Promotes proinflammatory cytokine expression, contributing to pathogenesis of acne	[53, 54]
Acetate	SCFA (FFA), SM	59.04	Breakdown of triglycerides on skin or dietary fibres in gut. Produced by many gut and skin bacteria	Influences differentiation of CD4^+^ cells such as Th1, Th17 and Tregs	[55]
Indole‐3‐propionic acid	Tryptophan derivative, SM	189.21	Tryptophan metabolism by gut bacteria and *Lactobacillus*	Antioxidant properties and helps modulate inflammation	[56, 57]
Indole‐3‐aldehyde	Tryptophan/derivative, SM	145.16	Tryptophan metabolism by gut bacteria and *Lactobacillus ruteri*	Topical application regulates inflammation and improves AD	[58, 59]
Indole‐3‐lactic acid	Tryptophan/derivative, SM	189.19	Tryptophan metabolism by gut bacteria and *Lactiplantibacillus* species	Topical application supresses AD symptoms	[60, 61]
Quinolinic acid	Tryptophan/derivative, SM	167.12	Tryptophan metabolism by skin microbiome, particularly *Bacteroidetes*	Lower concentrations found in psoriasis patients; topical application alleviates symptoms	[62]
Bacteriocins	Ribosomally synthesised from amino acids, some are a type of SM	~3000–10 000+	Produced by many skin bacteria such as *Staphylococci* and *C. acnes*	Antimicrobial and regulate the skin microbiome	[28, 63]
Lantibiotics	Ribosomally synthesised and post‐translationally modified peptides	> 5000	Produced by several skin bacteria. Most notable is nisin, produced by *Lactococcus lactis*	Antimicrobial activity against many Gram‐positive pathogens	[64, 65]
Ectoine	Amino acid derivative, SM	142.16	Produced by halophiles	Cell protector that increases skin hydration and reduces TEWL. Decreases inflammation in inflammatory skin disorders	[66, 67]

Stenz et al. [47] reported that oleic acid (a free fatty acid [FFA]), a metabolite produced by several microorganisms found on the skin [48, 68], inhibited the adhesion and biofilm formation of multiple strains of *S. aureus*. Similarly, sapienic acid was also found to inhibit *S. aureus* and *S. epidermidis* in vitro [69]. Nisin, a type of lantibiotic, has been shown to improve *S. aureus* induced skin conditions in mice due to its antimicrobial activity against Gram‐positive species [64]. Ectoine has been shown to increase barrier hydration and decrease TEWL. Furthermore, topical application has shown immunomodulatory effects in inflammatory skin conditions [66, 67]. Another mouse model reported that FFAs can lower the pH of the skin, which is known to increase the selection on microbial growth [70, 71]. FFAs metabolised from linoleic acid can inhibit keratinocyte proliferation by blocking the secretion of growth‐promoting cytokines [70, 72]. Additionally, the culture of FFAs (lauric acid, palmitic acid, or oleic acid) with sebocytes upregulates the expression of the AMP β‐defensin‐2, which can reduce the viability of *C. acnes* [73]. *Malassezia furfur*, a yeast commonly found on the skin, metabolises oleic or nonanoic acid into azelaic acid, an anti‐inflammatory and antimicrobial, beneficial for combating *C. acnes* in acneic skin [49, 50, 74, 75]. MMs also influence immune differentiation in vivo. Mucosal‐associated invariant T‐ (MAIT) cells, which present MMs to the wider immune system, were not produced in GF mice due to a lack of intermediates of vitamin B2 synthesis [76, 77, 78], increasing bioburden after infection [79].

MMs can also be pathogenic to the epidermal tissue. *C. acnes* can produce lipid derivatives (propionate and valerate), which promote proinflammatory cytokine expression in keratinocytes, contributing to the pathogenesis of acne [53]. Moreover, Johnson et al. [80] reported that acneic skin is colonised by a strain of *C. acnes* that produces higher levels of proinflammatory porphyrins compared to healthy skin, while Qiu et al. [81] found that propionate concentration in the sebum of AD patients was lower than healthy controls. The authors also found that AD in mice was relieved by topical application of propionate by inhibiting the IL‐33 pathway.

Due to their small size, metabolite‐host interactions may occur beyond the skin surface, into the dermis and subcutis, yet this remains to be investigated. One emerging area is that of the role of microbial tryptophan metabolites in skin barrier homeostasis, and this is discussed further below.

## 4 Tryptophan Metabolites and the Aryl Hydrocarbon Receptor

Tryptophan is an essential amino acid which is metabolised by many gut and skin commensals [82], the byproducts of which have been shown to have several benefits for epidermal health. Elias et al. [82] found that 14/18 bacterial skin isolates produced tryptophan metabolites, and their supernatants activated the AHR to varying extents, depending on the concentration and combination of metabolites present. The AHR is a ligand‐activated transcription factor and is present on many cell types. It has influence over genes for xenobiotic protection [83], keratinocyte differentiation [84], AMP production, and a range of cytokines [83, 85]. The complexity and breadth of AHR activation is highlighted by the numerous reviews published regarding its role in the gut, skin and disease [83, 85, 86, 87, 88]. Importantly, the AHR can be activated by a range of MMs, especially tryptophan metabolites [58, 89, 90, 91].

Uberoi et al. [22] observed that metabolites of tryptophan, particularly indole‐3‐aldehyde and indole acetic acid, were more abundant in FF50 mice (GF mice with an engineered microbiome containing 50 skin commensal bacteria) compared to GF mice. These metabolites (along with other tryptophan‐derived compounds) were shown to decrease TEWL in tape‐stripped mouse epidermis and enhance TEER in human keratinocytes. Higher doses of indole‐3‐aldehyde and indole acetic acid further increased TEER. These effects were shown to be mediated through AHR activation.

Lack of tryptophan metabolites is implicated in skin disease, with concentrations being lower in lesional and non‐lesional skin of AD patients. Topical addition of indole‐3‐aldehyde significantly improved AD symptoms in mice [58], while work in a human skin equivalent model showed that indole‐3‐lactic acid suppresses an induced AD‐like phenotype [60]. These studies point to the essential roles of tryptophan metabolites produced by the microbiome in skin homeostasis.

## 5 Does the Skin Microbiome Influence the Epidermal Response to UVR?

UVR is a significant challenge to the human skin, with excess exposure being associated with inflammation, ageing, and cancer [92, 93]. The effects of the microbiome on skin physiology in the context of UVR exposure have only just begun to be investigated. However, significant evidence from mouse models suggests that the microbiome regulates UVR‐induced inflammation and keratinocyte proliferation.

Patra et al. [3] reported that GF mice had increased mast cell and macrophage infiltration into the cutaneous tissue after UVR exposure compared to wild‐type (WT) mice. WT mice also had enhanced epidermal hyperplasia and neutrophilic infiltration compared to GF mice. In a continuation of this work, Patra et al. [2] found that the skin microbiome also regulates the host metabolome after UVR exposure, with GF and disinfected mice having a differential abundance of alanine, choline, glycine, glutamine, and histidine after a single dose of UVR compared to WT mice. The same group also reported that some skin commensals metabolise *cis‐*urocanic acid (a UVB photoproduct) to proliferate. This in turn reduces *cis‐*urocanic acid on the skin, inhibiting immune suppression; blocking this metabolism restored immune suppression [94]. The microbiome is also involved in regulating the expression of IL‐6 and TNF‐α following UVR exposure [20], while some skin commensals, such as *Micrococcus luteus* , express carotenoid pigments in response to UVR, which act as chromophores and exhibit antioxidant effects by scavenging reactive oxygen species [95, 96, 97]. As well as this, *C. acnes* can release porphyrins, which absorb UVR and protect bacterial cells and surrounding tissue from UVR [4, 98].

Additional evidence from photodermatoses highlights the role of the microbiome in the cutaneous response to UVR. Zarfl et al. [99] reported that the expression of 20 cytokines involved in apoptosis, inflammation, immune cell recruitment, cellular growth, and differentiation was significantly dysregulated in individuals with polymorphic light eruption (PLE) compared to healthy controls. Skin disinfection restored cytokine balance. Repeated UVR exposures exacerbated the difference in cytokine expression relative to controls. However, no significant variations were noted in erythema, pigmentation, or apoptosis.

The mechanisms underlying these findings require further investigation. However, there are several possibilities which we discuss below.

## 6 UVR Exposure Alters the Composition of the Skin Microbiome

The effects of UVR on bacteria have been well documented for decades, as UVR is widely recognised as an effective sterilisation method [100]. Consequently, it can be hypothesised that UVR impacts the skin microbiome, likely in a species‐specific manner. Some recent studies have explored these effects.

Burns et al. [5] examined the effects of UVR exposure in human volunteers. Cyanobacteria was reportedly differentially affected, being increased or decreased in abundance by UVA or UVB respectively, while a decrease in Lactobacillaceae and Pseudomonadaceae was also observed. Willmott et al. [1] observed that individuals who participated in ‘sun‐seeking’ holidays experienced a reduction in Proteobacteria compared to holidaymakers who avoided sunlight. This change in the microbiome was able to recover 28‐days post‐holiday. Harel et al. [101] compared the skin microbiomes of Mediterranean lifeguards (‘high‐UV’ group) and sun protected individuals. Microbiome composition between groups was similar in the winter months. However, in the summer they observed alterations in the microbiome composition of the high‐UV group, specifically in low abundance species, such as Planctomycetes and Cryomorphaceae. A study investigating PLE revealed that patients exhibited reduced microbial diversity, largely due to increased colonisation by pathogenic bacteria such as *S. aureus* . UVR exposure led to further declines in microbial diversity, accompanied by a loss of beneficial commensal species. The UVR sensitivity of these depleted commensals was later confirmed through in vitro experiments [102].

Changes in microbiome composition after UVR exposure may occur, for example, due to differential sensitivities of species to UVR. Matallana‐Surget et al. [103] demonstrated that prokaryotes with GC‐rich genomes are more susceptible to UVB. In contrast, some microbes show limited sensitivity to UVR. For example, UVB therapy in AD patients reduces *S. aureus* bioburden, while *S. epidermidis* is unaffected [104]. Prokaryotic defence mechanisms such as the production of porphyrins (by e.g., *C. acnes*) or carotenoid pigments (e.g., by *M. luteus* ) are mechanisms by which microorganisms could protect themselves from UVR [4]. These defence mechanisms are not ubiquitous in skin microbiota, which provides some organisms with an advantage over others following UVR exposure, thus potentially leading to a change in the overall composition of the skin microbiota.

## 7 UVR May Lead to Microbial Lysis

As previously discussed, UVR is an effective sterilisation method against bacteria. Its mutagenic effects can damage bacterial DNA, leading to cell death [105] which often results in lysis [106]. In addition, UVR is known to interrupt the structure of the lipid bilayer, increasing pore formation and permeability [107]. Lysis of skin commensal bacteria following UVR exposure has not been extensively studied. However, we hypothesise that the breakdown of microorganism structures as a result of UVR exposure could be one way in which the microbiome influences the epidermal response to UVR. UVR exposure can lead to the release of lipopolysaccharides, oleic acid, and porphyrins as well as a range of pathogen‐associated molecular patterns [4, 108]. These can interact with keratinocyte receptors such as the AHR and TLR2, known to influence a range of genes related to the immune and UVR response. Metabolites, including tryptophan metabolites, accumulate in the cytoplasm before secretion and can also be used for intracellular processes [109]. Therefore, cell lysis could increase the presence of MMs, known to enhance barrier integrity and attenuate inflammation. There is also evidence that microbial DNA photoproducts could be a potential trigger of the immune response [110].

Additionally, lysed bacterial cells release an array of fresh nutrients, potentially enriching the environment and providing new food sources for surrounding microorganisms. This could further alter the composition of the microbiome post‐UVR exposure by promoting the growth of surrounding microbes in a species‐specific manner.

## 8 UVR May Change Microbial Metabolism

UVR‐induced changes to the microbiome are likely to have a cascading impact on the metabolome, which interacts with host cells through various mechanisms. This hypothesis stems from the strong connection identified between the microbiome's composition and its metabolome. Many metabolites that are produced by skin microbes are synthesised from nutrients provided by the host or its surrounding microbiome [19]. UVR exposure is known to kill and fragment bacterial cells as well as cause functional changes to host cells, highlighting a potential mechanism by which the metabolome may be altered by UVR exposure.

Despite the limited literature, there is a strong association between the skin microbiome's composition and its metabolite profile, with different niches having distinct metabolomes. Roux et al. [111] found significant associations between microbial metabolome, topographical location, and microbiome composition. Moist acidic skin was linked to amino acids and tricarboxylic acids, while dry acidic skin was associated with long‐chain unsaturated fatty acids. In basic environments, phospholipids were linked to moist skin, and ceramides to dry skin. Specific microbes also showed strong associations with certain metabolites. Bouslimani et al. [112] found a correlation between the presence of *Cutibacterium* and the abundance of lipids, such as oleic acid, palmitic acid, and monoacylated glycerol's, as well as an association between tryptamine and *Staphylococcus*. This link between microbiome, metabolome, and topographical location on the skin most likely arises from two factors: the distinct association between topographical location and specific bacterial colonisation [113], and the differences in nutrients available at each niche.

## 9 Is the Skin and Its Microbial Metabolome Sensitive to Perturbations?

**FIGURE 1:**
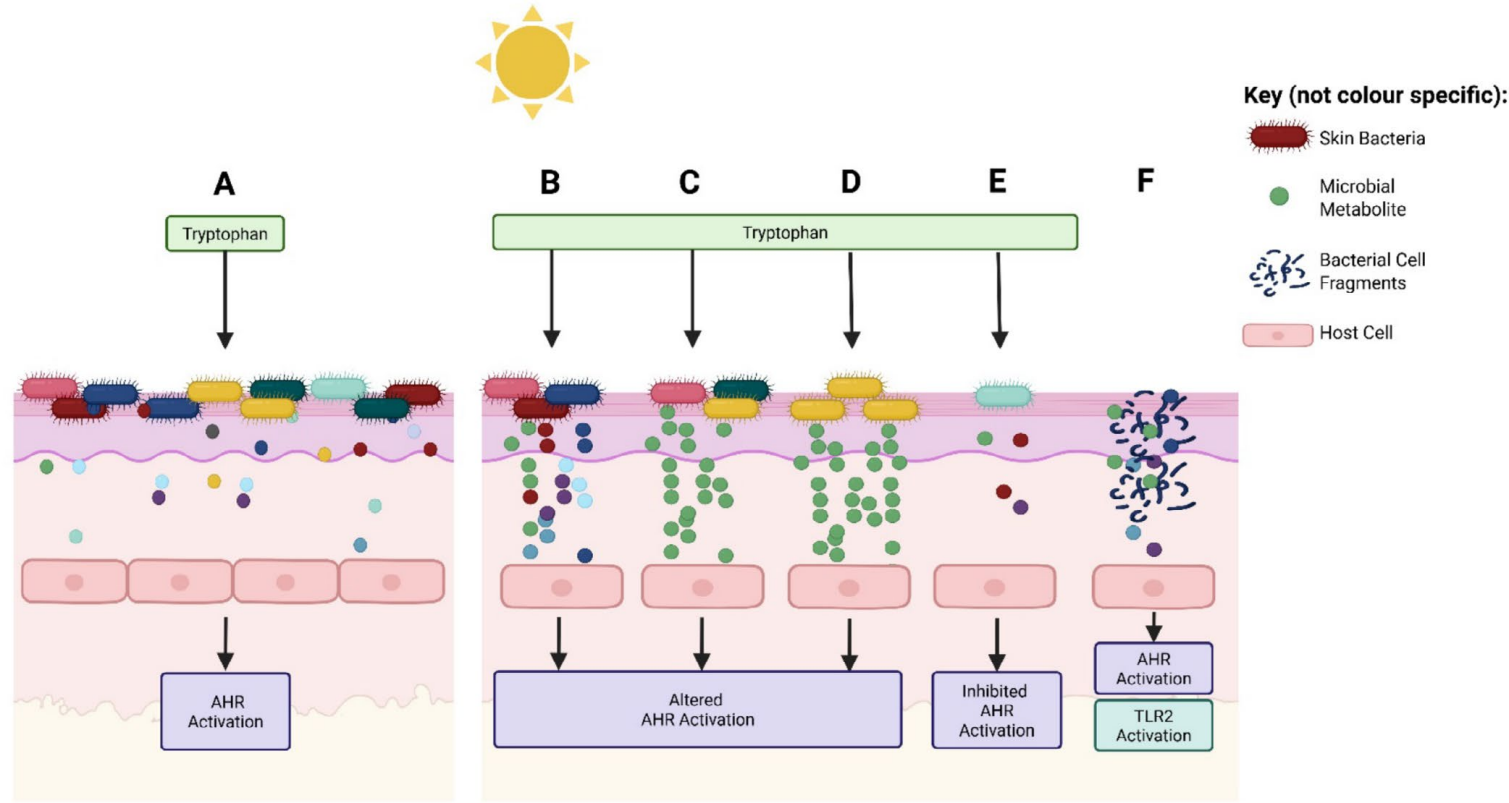
A diagram representing the potential effects of UVR on the skin microbiome and metabolome. (A) Microbiome and metabolites before exposure to UVR. (B‐F) Potential effects of UVR exposure. (B) Microbial metabolism could be increased (or decreased) by UVR. (C) Diversity of MMs could be altered by UVR exposure. (D) UVR exposure can influence microbiome composition, which may impact metabolome diversity. (E) UVR exposure can decrease microbial abundance, potentially leading to fewer metabolites. (F) UVR exposure could cause bacterial lysis, releasing material known to activate TLR2 and the AHR (including intracellular metabolites). Image made using Biorender.

There are several mechanisms by which UVR is likely to perturb the microbial metabolome (Figure 1. The link between topographical location, microbiome and metabolome is a key factor, highlighting that changes in microbiome composition following UVR exposure are likely to have a ‘knock on’ effect on the metabolome composition. In addition, UVR is directly antimicrobial and therefore reduced abundance is likely to affect the concentration of MMs. Due to the cross‐talk between the microbiome and host, it is also possible that functional changes to host cells following UVR exposure could alter microbial metabolism or the nutrients available for metabolism. Death to surrounding microbial cells after UVR exposure could also enrich, or offer alternative, nutrients available for metabolism. Finally, due to increases in oxidative stress and DNA damage caused by UVR, it is possible that microbial metabolism may be directly affected by irradiation.

Although there is limited evidence to support these hypotheses, there is some evidence that UVR can directly alter microbial metabolism. Chen et al. [114] found that UVR decreased microbial diversity in wastewater following UVR exposure. Furthermore, amino acid, lipid, terpenoid, and polyketide metabolism was inhibited by irradiation, and the expression of genes involved in nitrogen metabolism was reduced with increased UVR exposure time, demonstrating the influence of UVR on a range of metabolic pathways in bacteria.

In addition, mycosporine‐like amino acid (MAA) production increases after UVR exposure, demonstrating its influence on microbial metabolism. These SM can absorb the photonic energies of UVR and convert this to heat without generating reactive oxygen species [115, 116, 117].

## 10 Future Perspectives

Excess UVR exposure is known to cause inflammation, as well as alter keratinocyte differentiation and the cutaneous immune system. Skin commensal microorganisms can produce metabolites that are known to influence the AHR, a receptor with influence over genes related to the UVR response. Therefore, understanding the relationship between UVR, the skin, and its microbiome presents a unique opportunity to advance our scientific knowledge and therapeutic innovation. We suggest that future investigations aiming to unravel the interactions between the skin and its microbiome, or to understand the role of UVR on the skin microbiome and its impact on the epidermal response, should examine microbial taxa and metabolites in tandem. Advances in metabolomics and DNA sequencing techniques provide promising avenues to explore these interactions in detail.

Current insights into UV–microbiome interactions largely come from human studies, which, while physiologically relevant, are limited by high inter‐individual variability, environmental confounders, and ethical constraints related to UVR exposure [118]. Mouse models are also commonly used but lack key anatomical and physiological features of human skin, reducing translatability. Ex vivo skin models offer an alternative, particularly when paired with defined microbiome models such as SkinCom [119]. However, detecting specific metabolites in small biopsies remains challenging due to their low concentrations, limitations in sampling techniques, and finite sensitivity of LC–MS methods. Despite these challenges, emerging studies continue to expand our understanding of the metabolome and the role of microbial metabolites in skin responses to UVR exposure.

Altered microbial metabolism, potentially induced by UVR exposure, may result in the production of metabolites not typically found in the skin microbiome, as well as changes in the concentration of existing metabolites. These shifts in metabolite profiles could create new opportunities for cosmetic and therapeutic applications, either by harnessing the production of novel metabolites or compensating for the loss of reduced metabolites. Additionally, understanding the role of MMs in UVR‐induced inflammation, immune modulation, and skin barrier integrity could drive innovation in dermatological therapies. For example, identifying microbiome‐derived metabolites that promote photoprotection or mitigate UVR‐induced damage may lead to the development of next‐generation sun care products. These products could go beyond traditional UV filters, incorporating probiotics, postbiotics, or metabolite‐inspired compounds designed to preserve or restore microbial balance following UVR exposure.

Furthermore, this emerging knowledge holds significant promise for managing photodermatoses. By targeting microbial dysbiosis or harnessing beneficial microbial metabolites, it may become possible to develop therapies that address the root causes of UVR sensitivity, rather than focusing solely on symptomatic relief.

## Author Contributions

Writing original draft and editing: S.D.M. Editing, project design and funding acquisition: A.J.M. Writing original draft, editing, project design and funding acquisition: C.O.

## Conflicts of Interest

The authors declare no conflicts of interest.

## Data Availability

The authors have nothing to report.
